# Acute psychosis as an initial manifestation of hypothyroidism: a case report

**DOI:** 10.1186/s13256-015-0744-z

**Published:** 2015-11-17

**Authors:** Shinichi Ueno, Satoko Tsuboi, Motoki Fujimaki, Hiroto Eguchi, Yutaka Machida, Nobutaka Hattori, Hideto Miwa

**Affiliations:** 1 Department of Neurology, Juntendo University Nerima Hospital, 3-1-10 Takanodai, Nerima, Tokyo 177-8521 Japan; 2 Department of Neurology, Juntendo University School of Medicine, 2-1-1 Hongo, Bunkyo, Tokyo 113-8421 Japan

**Keywords:** Myxedema madness, Hypothyroidism, Psychosis, L-thyroxine

## Abstract

**Introduction:**

Hypothyroidism is one of the most important causes of treatable dementia, and psychosis occasionally associated with it is known as *myxedema madness*. We report a case of a 90-year-old patient who developed myxedema madness acutely without overt clinical symptoms and signs suggestive of hypothyroidism.

**Case presentation:**

A 90-year-old Japanese man, a general practitioner, was admitted to our emergency room because of acute-onset lethargy, delusions, and hallucinations. He had been actively working until 3 days before the admission. Upon admission, his general physical examination was unremarkable. However, a blood investigation showed the presence of hypothyroidism, and computed tomography revealed pleural effusion and ascites. Electroencephalography revealed diffuse slow waves with a decrease of α-wave activity. A single-photon emission computed tomography scan revealed a decrease of cerebral blood flow in both frontal lobes. The patient was soon treated with thyroid hormone replacement therapy. Following normalization of his thyroid function, both pleural effusion and ascites diminished and his electroencephalographic activity improved simultaneously; however, he did not recover from his psychosis.

**Conclusions:**

Myxedema madness should be kept in mind in the differential diagnosis of acute psychosis in elderly patients, particularly the oldest patients as in our case, because manifestations of hypothyroidism often may be indistinguishable from the aging process.

## Introduction

Hypothyroidism is the most common hormonal disorder, but it is by no means easy to make an appropriate diagnosis in elderly patients, because thyroid-associated symptoms are very similar to symptoms of the normal aging process [1]. Psychosis occasionally occurs in patients with hypothyroidism, in whom it is known as *myxedema madness* [2]. Recently, we encountered an oldest old patient with acute psychosis without overt physical symptoms and signs suggestive of hypothyroidism.

## Case presentation

A 90-year-old Japanese man was admitted to our hospital because of lethargy with a suspiciously acute onset. He had been actively working as a general practitioner until 3 days before the admission. His past history was unremarkable, except for hypertension and prostatic hypertrophy. He had no evidence of recent falls. He had no family history of psychiatric disease. For 2 days before the admission, he repeated to himself an inconsistent story that did not make sense to his family members, and he talked toward the wall as if someone were there. He was drowsy but suddenly sang cheerfully, although he usually did not like singing.

Upon admission (day 0), his physical examination revealed no remarkable findings, except mild hypertension. His blood pressure was 160/100 mmHg, his body temperature was 36.3 °C, and his pulse was 60 beats/minute. There were no findings of dry skin, hoarseness, nonpitting edema or other obvious symptoms. His mental status examination revealed disturbance of consciousness, concentration, and memory. It was difficult to make an accurate assessment of his cognitive function because he had difficulty in continuously responding to questions. In his neurological examination, he was found to be somnolent and uncooperative with a detailed interview, and he also exhibited delirium by carrying on a monologue as if speaking to someone. He could not correctly answer questions to assess orientation and cognitive function, mistaking the place and date. It also was difficult to evaluate him based on a cognitive function scale. His neurological examination also revealed hearing loss, gait disturbance with small steps and hesitation, no sensory disturbance, and an obvious difference in bilateral muscle strength between the limbs. Blood investigations revealed a thyroid-stimulating hormone (TSH) of 105.87 mIU/ml (normal range 0.54–4.26 mIU/ml) and free thyroxine (T4) of 0.34 ng/dl (normal range 0.71–1.52 ng/dl). His thyroid peroxidase antibodies and thyroglobulin antibodies were negative. Cerebrospinal fluid analysis revealed a normal cell count but mild protein level elevation (58.5 mg/dl).

Computed tomography of the patient’s whole body revealed pleural effusion on the left side and a moderate amount of ascites. Magnetic resonance imaging of his head revealed crescent-shaped foci of T2 hyperintensity visualized as slight effusion below the dura mater, which was considered brain atrophy. Electroencephalography (EEG) revealed slow α- and θ-waves. *N*-isopropyl-p-(iodine-123)-iodoamphetamine single-photon emission computed tomography (IMP-SPECT) revealed a decrease of cerebral blood flow in both frontal lobes. We noted that the patient had no organic brain disease and no obvious metabolic abnormalities such as vitamins. He had a high TSH level and a low T4 level, so we diagnosed him with psychosis and disturbance of consciousness with hypothyroidism. For this condition, about 10 days after his admission, we immediately started oral therapy with thyroid hormone replacement (L-thyroxine 50 μg/day). Thyroid testing showed a decreased TSH level to 9.53 mIU/ml and elevated free thyroxine of 1.34 ng/dl after administering an L-thyroxine dose up to 75 μg daily, but his psychotic symptoms did not improve soon thereafter. Following normalization of his thyroid function, both pleural effusion and ascites diminished. Simultaneously, his EEG activity normalized with normal α-wave activity. For 2 months after onset, his consciousness level gradually improved, and he became fully awake; however, his hallucinations and deliriums still persisted. To control his psychotic symptoms, risperidone (0.5 mg/day) was added to his regimen because, as he was elderly, we were concerned about side effects such as hypersomnia. The second IMP-SPECT scan results remained abnormal, with decreased function in his bilateral frontal regions. Fifty days after his admission to our hospital, he was referred to another hospital for rehabilitation because of a decline in his muscle strength due to wasting.

## Discussion

One of the most remarkable clinical characteristics of our patient is that the initial manifestation of hypothyroidism was acute psychosis. The fact that he had been working actively as a physician 3 days before his admission indicates that no prominent deterioration had been present in his mental and physical activities and that frontotemporal dementia does not in general have an acute onset. Although numerous symptoms and signs associated with hypothyroidism are known to appear in these patients [1, 3], patients with hypothyroidism do not always exhibit typical clinical manifestations [4]. Diagnosis of hypothyroidism in elderly people is often difficult because a variety of symptoms are considered to be normal parts of the aging process [1]. Particularly, in the oldest old patients, such as ours, who was older than 90 years of age, it is by no means easy to make a prompt diagnosis of hypothyroidism.

Psychosis associated with myxedema has been termed *myxedema madness* since it was first described in 1949 [2]. In the older literature, delusions and hallucinations associated with hypothyroidism were described as emerging after the onset of physical symptoms, often after a period of years or months [5]. However, it has recently been reported that no correlation appears to exist between the degree of thyroid dysfunction and psychiatric symptoms that subsequently develop [6]. In our patient, physical findings suggestive of the presence of hypothyroidism, which include edema or dry skin, were not prominent. Both pleural effusion and ascites were only the latent symptoms, and both of them disappeared after initiation of thyroid hormone replacement therapy, supporting that the patient’s symptoms were caused by thyroid hormone deficiency. These findings may implicate that psychosis can appear, as in our present patient, even if patients have no severe physical symptoms of hypothyroidism.

As has been suggested, psychiatric symptoms often do not respond well to thyroid replacement therapy, regardless of whether physical symptoms improve [7]. Similarly, in our patient, although his lethargy and drowsiness improved after thyroid replacement therapy, his delusions continued, although he had no obvious other metabolic disorders. This may be because, as we postulated, chronic metabolic damage may damage irreversible changes in the central nervous system [8]. It was recently reported that, based on positron emission tomography data, cerebral blood flow and glucose metabolism were chronically reduced in severe hypothyroidism [8]. Indeed, in our patient, a decrease of cerebral blood flow in his bilateral frontal lobes did not improve after thyroid replacement therapy.

## Conclusions

Hypothyroidism is the most common hormonal disorder, but it is by no means easy to make an appropriate diagnosis in elderly patients. Although numerous symptoms and signs are known to appear that are associated with hypothyroidism, we propose that acute myxedema madness should be kept in mind in the differential diagnosis of acute psychosis in elderly patients, particularly in the oldest old patients, because they may have treatable dementia and psychosis.

## Consent

Written informed consent was obtained from the patient for publication of this case report and any accompanying images. A copy of the written consent is available for review by the Editor-in-Chief of this journal.

## Abbreviations

CSF: cerebrospinal fluid
EEG: electroencephalography
IMP-SPECT: *N*-isopropyl-p-(iodine-123)-iodoamphetamine
T4: thyroxine
TSH: thyroid-stimulating hormone
